# Study of the Stability of Wine Samples for ^1^H-NMR Metabolomic Profile Analysis through Chemometrics Methods

**DOI:** 10.3390/molecules28165962

**Published:** 2023-08-09

**Authors:** Martha E. García-Aguilera, Ronna Delgado-Altamirano, Nayelli Villalón, Francisco Ruiz-Terán, Mariana M. García-Garnica, Irán Ocaña-Ríos, Eduardo Rodríguez de San Miguel, Nuria Esturau-Escofet

**Affiliations:** 1 Instituto de Química, Universidad Nacional Autónoma de México, Ciudad de Mexico 04510, Mexico; mgarciaa@iquimica.unam.mx (M.E.G.-A.); ti2dronna@hotmail.com (R.D.-A.); lorienaule@gmail.com (N.V.); mariana.mishellgg@gmail.com (M.M.G.-G.); ior84bd@gmail.com (I.O.-R.); 2 Facultad de Química, Universidad Nacional Autónoma de México, Ciudad de Mexico 04510, Mexico; panchote@unam.mx

**Keywords:** red wine, ^1^H-NMR, temperature–time changes, PCA, ASCA, PARAFAC

## Abstract

Wine is a temperature, light, and oxygen-sensitive product, so its physicochemical characteristics can be modified by variations in temperature and time when samples are either sampled, transported, and/or analyzed. These changes can alter its metabolomic fingerprinting, impacting further classification tasks and quality/quantitative analyses. For these reasons, the aim of this work is to compare and analyze the information obtained by different chemometric methods used in a complementary form (PCA, ASCA, and PARAFAC) to study ^1^H-NMR spectra variations of four red wine samples kept at different temperatures and time lapses. In conjunction, distinctive changes in the spectra are satisfactorily tracked with each chemometric method. The chemometric analyses reveal variations related to the wine sample, temperature, and time, as well as the interactions among these factors. Moreover, the magnitude and statistical significance of the effects are satisfactorily accounted for by ASCA, while the time-related effects variations are encountered by PARAFAC modeling. Acetaldehyde, formic acid, polyphenols, carbohydrates, lactic acid, ethyl lactate, methanol, choline, succinic acid, proline, acetoin, acetic acid, 1,3-propanediol, isopentanol, and some amino acids are identified as some of the metabolites which present the most important variations.

## 1. Introduction

Wine is a fermented alcoholic beverage extensively distributed, appreciated, and investigated around the world. Chemical characterization of wine has evidenced the complexity of this matrix since it is composed of a high quantity and diversity of metabolites (also known as metabolome). Metabolomics is one of the most recent omics sciences, and it comprises the study of the metabolome of organisms, biological systems, or products in a specific condition. Liquid chromatography–mass spectrometry (LC–MS) and proton nuclear magnetic resonance (^1^H-NMR) are two analytical platforms used for metabolomics. Although LC–MS is very sensitive and possesses the ability to quantify a greater number of compounds, ^1^H-NMR is advantageous since it is non-destructive, robust, highly reproducible, requires short analysis time, and laborious sample preparation steps are not needed. In addition, it has the capability of the detection of diverse organic compounds and supplies very specific structural information. For this reason, it has been widely used for wine fingerprinting and metabolomics [1,2,3]. Correlation of the ^1^H-NMR spectra data to some properties of wine using chemometrics methods can help to find patterns of similarity or differences according to vineyard [2], origin [3], variety [4], vintage and ageing [5,6,7], bottle aging [8], quality control [2], authentication [9], color evolution, and stability [10], and cultivation practices [5,9,11], among other factors or their combination [8,12,13,14]. Chemometric methods make use of multivariate analysis (MVA) techniques. 

In recent years, many studies have been conducted using unsupervised methods such as principal component analysis (PCA) and supervised methods such as partial least squares discriminant analysis (PLS-DA) and/or its orthogonal form (OPLS-DA) to analyze the sources of variation and other underlying factors, as previously mentioned. In addition, techniques such as hierarchical cluster analysis (HCA) and linear discriminant analysis (LDA) have also been used to predict geographical origin [3,15] and variety [16,17,18]. Nevertheless, these chemometric methods do not consider the explicit inclusion of temporal variation during modeling.

Although wine is a relatively stable product while it is in the bottle, as soon as it is opened and poured into different recipients, either for drinking or chemical analysis, air promotes reactions that affect its characteristics, giving a continuously changing dynamic system. Samples taken from wineries and transported at room temperature are subjected to strong variation as well, especially if long transport distances are considered. In this regard, chemometrics investigation of gas chromatography (GC) data and enological parameters (absorbance at 420 nm, free SO_2_, total SO_2_, total phenol, and total aldehyde) of a bag-in-box white wine stored at different temperatures and times showed a grouping trend that was influenced by both variables. In addition, the maximum storage time could be predicted accurately by partial least squares (PLS) regression of the GC data [19]. In another study, hierarchical and non-hierarchical cluster analyses were employed to monitor the level of biogenic amines in opened bottles against time and other conditions such as different temperatures and stopper type (screw cap or cork), and use of vacuum devices by dispersive liquid–liquid microextraction–gas chromatography–mass spectrometry (ME–GC–MS) to reveal latent relationships between wine brands, the conditions for their storage, and their amine content [20]. Furthermore, wine evolution during bottle aging has also been studied by ^1^H-NMR spectroscopy and PCA [8]. The authors found that metabolite variations due to wine aging were minimal compared to those that resulted from a different wine type and wine geographical origin. Storage at a low and controlled temperature for 2 or 4 years allowed for identifying a decrease in organic acids (lactic acid, succinic acid, and tartaric acid) and an increase in esters (ethyl acetate and ethyl lactate) content for most wines. Catechin and epicatechin decreased during aging in all wines, while gallic acid increased in almost all red wines [8]. The influence of transport temperature profiles on wine quality was also studied by simulating transport conditions in a climate chamber for five wines representing international wine styles: one sparkling, two white, and two red still wines. Analytical and sensory results demonstrated that a significant temperature influence, calculated as temperature–time equivalence, needs to be reached before quality effects are observed. The wines of a fruity and lighter style were more sensitive than the wines with higher alcohol and wooden aging. Furthermore, except for Champagne, sensory examinations showed a regular linear trend with exposure severity starting at the time equivalence at 15 °C. The sensory results correlated well with the ultraviolet–visible (UV–vis) and head-space solid-phase microextraction gas-chromatography mass-spectrometry (HS-SPME-GC–MS) results [21].

Metabolomic analysis of wines requires well-traceable samples; however, these samples may have different origins and could be sampled in different forms, e.g., they can be taken directly from cellars using either plastic or glass containers or they can come from a sampling of commercial bottles from restaurants/bars, etc., by regulatory authorities in the interest of public health and safety, to be transported to laboratories. No matter the procedure, the storage and transport conditions may impact the future analysis of the samples and, consequently, the results and conclusions of the investigation. As no work has been previously found that evaluates the effects of temperature, time, and their interaction using complementary chemometrics methods, neither to consider the experimental design information in the chemometric evaluation, this work aims to compare and analyze the information obtained by different chemometric methods used in a complementary form (PCA, ANOVA simultaneous component analysis (ASCA), and parallel factor analysis (PARAFAC)) to study ^1^H-NMR spectra variations of red wine samples kept at different temperatures and time lapses since the bottles were opened. Through these multivariate analyses, the identification of relevant characteristics and changes that permits deciding about the sample conditions before further metabolomic studies is attained.

## 2. Results and Discussion

### 2.1. NMR Fingerprint

**Figure 1:**
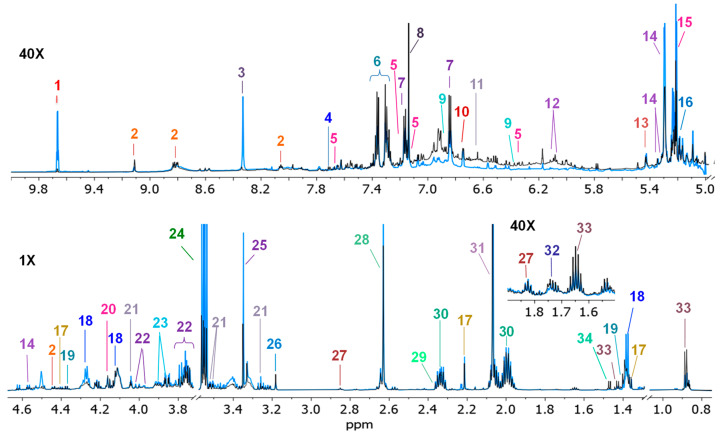
Overlayed ^1^H-NMR spectra (700 MHz, D_2_O, 300 K) of S2 wine sample at “zero-time” (black) and stored at 40°C for 67 days (blue). Identified compounds are numbered and listed in Table 1.

Representative ^1^H-NMR spectra of wine taken as soon as the bottle was opened and after long-time storage at extreme temperatures (67 days at 40 °C) are shown in Figure 1. Thirty-five compounds were identified, and changes in the intensity of some signals were detected as the main differences between both spectra, e.g., the intensity of signals from acetaldehyde (*δ* 9.67, q), formic acid (*δ* 8.33, s), succinic acid (*δ* 2.63, s), and acetic acid (*δ* 2.07, s), was increased. All the metabolites identified in the spectra with their chemical shift (*δ*, ppm), multiplicity, and constant coupling (*J*, Hz) are shown in Table 1. It is important to note that as no buffer was used in sample preparation, the principal consequence may be the changes in chemical shifts in some signals. For example, succinic acid singlet presented variations around 1.24 Hz after 1 day of storage at 40 °C, indicating possible pH changes in the sample. 

### 2.2. Chemometric Analyses

#### 2.2.1. Principal Component Analysis (PCA)

**Figure 2:**
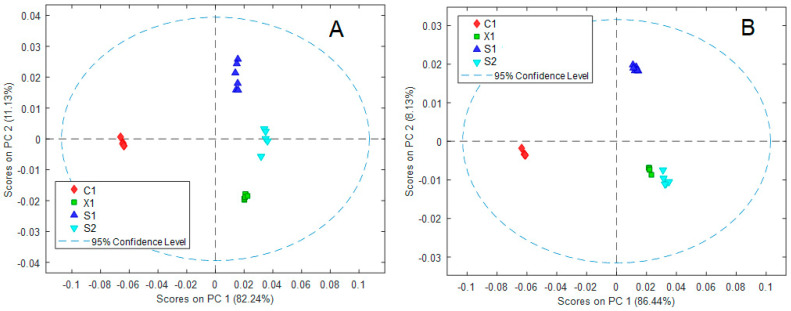
Score plot of 3 control samples of each wine (C1, X1, S1, and S2) randomly acquired by duplicate over a maximum time of 10 h. (**A**) considering all spectra and (**B**) eliminating three regions (4.60–4.46, 4.34–4.24, and 1.41–1.36 ppm).

**Figure 3:**
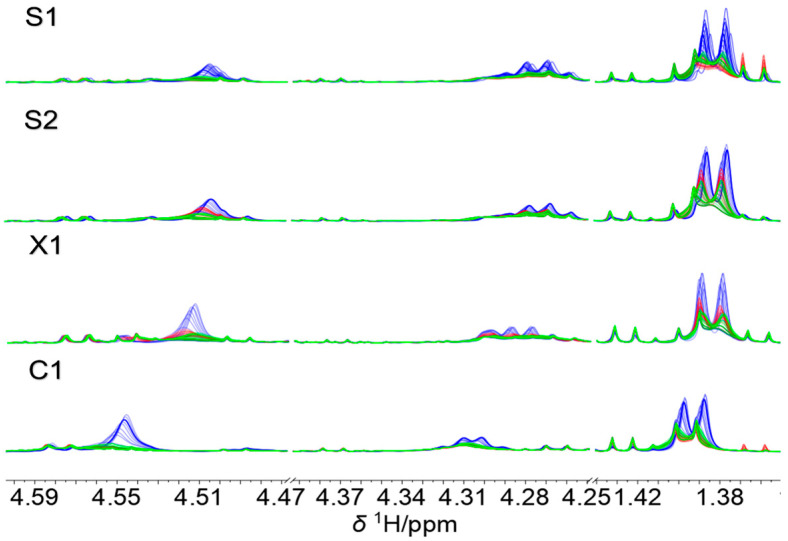
Expansion of three regions in the overlapped ^1^H-NMR spectra (700 MHz, D_2_O, 300 K). Samples S1, S2, X1, and C1, respectively, stored at 4 °C (green), 20 °C (red), and 40 °C (blue), for 67 days.

The ^1^H-NMR spectra of three control samples of each wine were randomly acquired by duplicate over a maximum time of 10 h to simulate normal variations during workday conditions. Then, ^1^H-NMR data were subjected to an exploratory PCA analysis showing time-course changes between controls and replicas (Figure 2A). The result of a three-component model provided a root mean square error of calibration (RMSEC) = 2.81 × 10^−4^ and a root mean square error of cross-validation (RMSECV) = 4.49 × 10^−4^ values. As some dispersion of data was observed, the PCA loading plot was analyzed, revealing that the regions in the spectra contributing to such a phenomenon were those associated with signals near to *δ* 1.38, *δ* 4.32, and *δ* 4.54 ppm. It was noticed that these regions corresponding to broad signals appeared even twenty minutes after the initial (zero-time) measure. For this reason, a complementary PCA was run, removing the regions 4.60–4.46, 4.34–4.24, and 1.41–1.36 ppm. As a result, a reduction of the dispersion between samples was observed (Figure 2B), although the difference between samples was conserved. To make these signals clearer, the expansions of the spectra regions with those chemical shifts for all samples (stored at all temperatures and times) are shown in Figure 3. These signals could not be assigned, but their analyses over all stored samples showed that they increased and were less wide through time and could be attributed to a degradation and oxidation process. For this reason, further analyses were performed keeping these regions.

The spectra variations of wine samples subjected to different temperatures and times were then analyzed by PCA. Briefly, models with 5–8 components were satisfactorily explained by 97.64 to 99.56% of spectra variation with RMSEC and RMSECV values between 0.0042–0.0068 and 0.0116–0.0145, respectively. No outliers were observed in the Hotelling T^2^ reduced vs. Q residuals reduced plots, indicating that all samples could be considered in the analysis.

**Figure 4:**
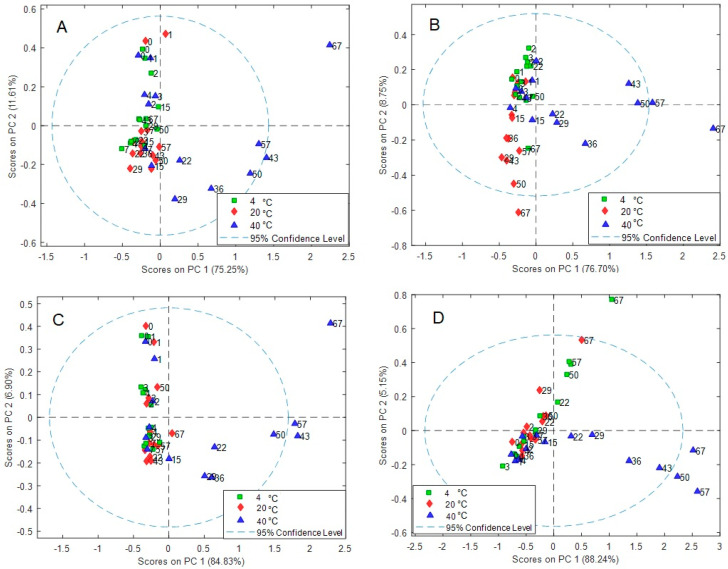
PCA score plots of wine samples at different times (0–67 days, numbers) and temperatures (4, 20 and 40 °C). (**A**) X1, (**B**) C1, (**C**) S1, and (**D**) S2. Time courses are indicated by the numbers inside the plots.

**Figure 5:**
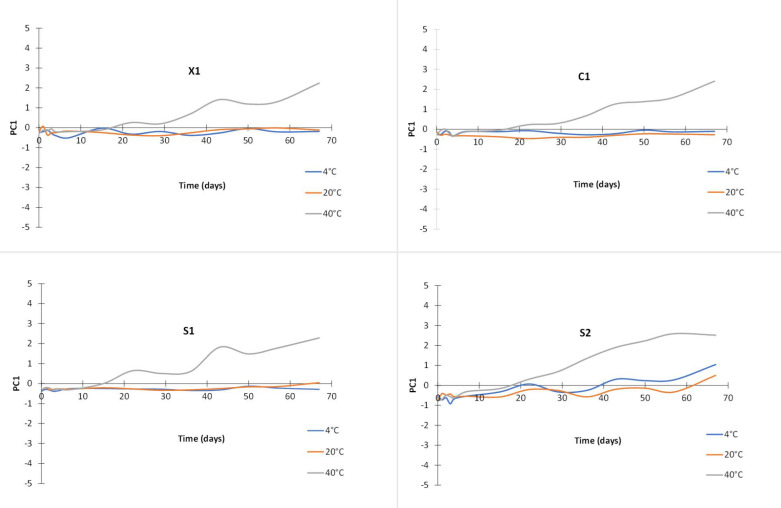
Time series plots of the PC1 scores at different temperatures for the different cases (X1, C1, S1, and S2).

In Figure 4, the PCA score plots of the models are shown. Clearly, spectra variations related to temperature and time are evidenced. In addition, some time-ordering of the samples is observed, indicating a gradual change of the spectra features through the elapsed time. The differences in the observed patterns comparing each case (C1, X1, S1, and S2) indicate that temperature and time effects are case-dependent, and the effect of temperature is not equal at each level of the time variable. In addition, it was observed that two components accounted for the spectra variations within 85.46 to 93.39%. To provide insight into the effects of time and temperature in each case, the time series plots of the score values of the first principal components are plotted in Figure 5, as ultimately, these terms account for 75.25–88.24% of the spectra variations in the data. As observed, PC1 accounts simultaneously for both effects, making the isolation of each source of variation indiscernible. Furthermore, as previously discussed, it is clearly observed that the effects vary within the cases because the score values present different profiles among them, showing the most different behavior in case S2. In addition, samples at 40 °C show the most significant variations in all cases, and the scores at 4 °C are very close to those at 20 °C in three cases, except for S2.

**Figure 6:**
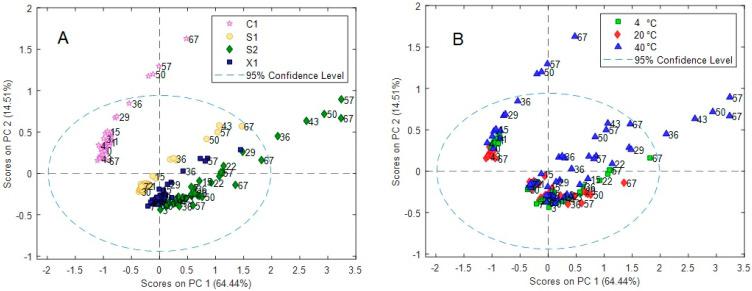
PCA score plot according to (**A**) case (C1, X1, S1, and S2) and (**B**) temperature. Time (0–67 days) courses are indicated by the numbers inside the plots.

Considering the similarity of the loadings for the components, it was decided then to analyze the four wines at once in one PCA analysis (Figure 6). The results of this procedure showed that some samples lay in the region of high leverage and outside the models’ plane in the Hotelling T^2^ reduced vs. Q residuals reduced plots; however, as they correspond to those with the longer times (67 days), it was decided to keep them during modeling. A model with nine components satisfactorily explained 98.42% of spectra variation with RMSEC and RMSECV values of 0.0082 and 0.0151, respectively.

The PCA scores plot of the model showing the data according to characteristic information, i.e., case (C1, X1, S1, S2), temperature (with colors), and time (with numbers), are shown in Figure 6. Changes in temperature and time are observed, where the wines at the lowest temperature vary the least. The differences in patterns observed indicate that the temperature and time effects are case-dependent and that the effect of temperature is not equal at each level of the time variable, i.e., interactions between both variables are relevant. Interestingly, comparing all data, the C1 case samples are present as a well-defined group in the graph (Figure 6A), indicating a well-differentiated pattern. More important, the effect of time seems to be more discernable than that of temperature (Figure 6B), and as time elapses, the data are more dispersed. This was confirmed in the Hotelling T^2^ reduced vs. Q residuals reduced plot, where samples at 57 and 67 days seem to be anomalous from the rest. Because PCA is not able to disclose in an independent way both effects, since no information related to the experimental design is included in the analysis, further studies were performed using the ASCA method taking advantage of the full-factorial structure of the experimental data matrix.

#### 2.2.2. ANOVA Simultaneous Component Analysis (ASCA)

Since the experiments were planned as a full-factorial design, an improvement in data interpretation with respect to PCA can be achieved by segmenting the information of the data matrix through ANOVA analysis prior to PCA. As the results in the previous section clearly indicated interactions among temperature, time, and case, a model contemplating up to two-variable interactions in a three-way ANOVA was considered. During modeling, *p*-values were obtained via 1000 permutation tests. Not surprisingly, the results of the analyses indicated that all variables play an important role as main effects and as interaction terms in all possible binary combinations at the 99.9% confidence level. Almost half (53.40%) of variability is due to differences among cases; temperature and time as the main effects have similar contributions (13.48 and 10.97%), while the contribution of the interactions follows the order: temperature × time (10.03%) > time × case (6.07%) > temperature × case (1.88%). 

**Figure 7:**
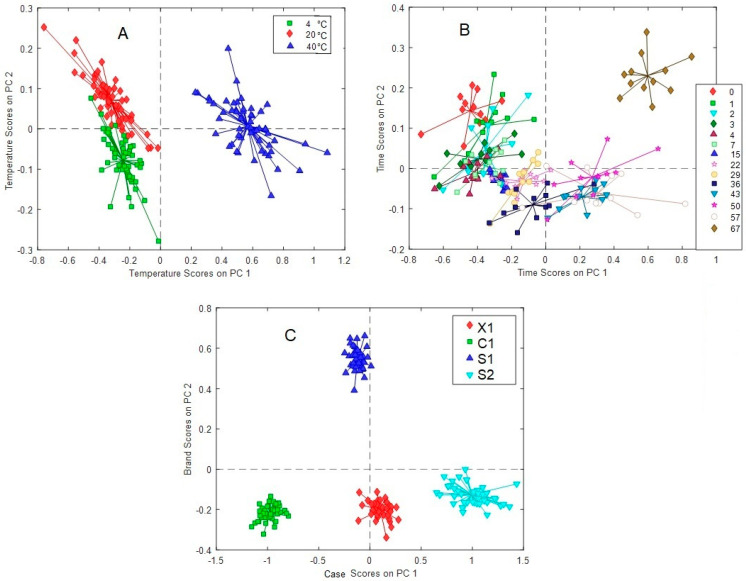
ASCA score plot of all wines for (**A**) temperature, (**B**) time, and (**C**) case effects.

In Figure 7, the score plots of the factors are displayed. Clearly, the separation of the different contributions by ANOVA allowed a better definition of groups (temperature, time, and case (C1, X1, S1, and S2)) in comparison to PCA. In Figure 7A, it is noted that temperature clusters the wines in practically two well-defined groups along PC1 since the scores at 40 °C are well separated from those at 4 and 20 °C, which are less different. Additionally, some distinction is observed along the PC2 for the latter samples. This is slightly observed for time (Figure 7B), where some trend is perceived as time increases ongoing from the first to the last days. On day 67, the spectra profiles considerably differ. More interestingly, a strong distinction according to the case (C1, X1, S1, and S2) is attained by the ASCA algorithm, which clearly isolates this contribution (Figure 7C), as was previously observed in PCA but not in a so-defined form. According to this, these four wine samples can be differentiated using only the PC1 and PC2 score values. Further confirmation of this result may allow us to define a characteristic signature according to the case, which may be helpful for identification purposes in conditions where the samples have been subject to temperature and temporal variations.

On resume, ASCA analysis clearly confirmed: (i) the importance of the interaction between temperature and time in a quantitative way, denoting that the main and interactions effects have comparable magnitudes; (ii) that samples at 4° and 20 °C are more similar between them in comparison to 40 °C; (iii) that a characteristic profile is present in each case. However, it was still difficult to isolate the contribution of the temporal variable to characterize the pattern followed by the spectra variations associated with this effect (Figure 7B). For this reason, the PARAFAC chemometric method was applied in the following section.

#### 2.2.3. Parallel Factor Analysis (PARAFAC)

**Figure 8:**
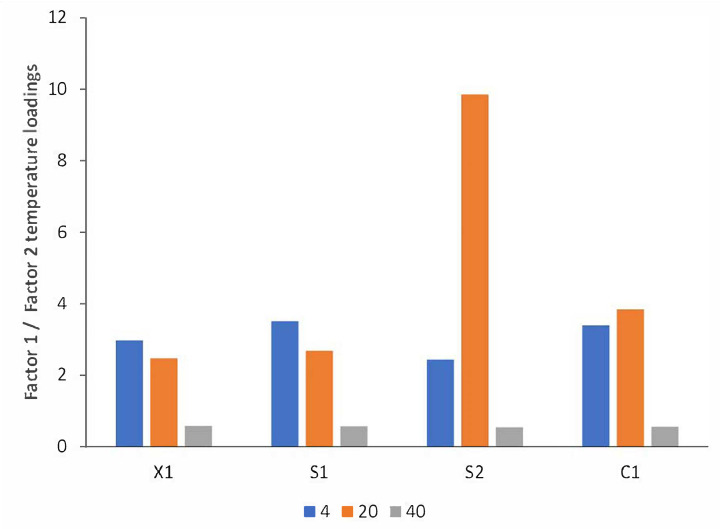
Temperature-mode PARAFAC loadings for the first to the second-factor ratio for the X1, C1, S1, and S2 cases at 4, 20, and 40 °C.

**Figure 9:**
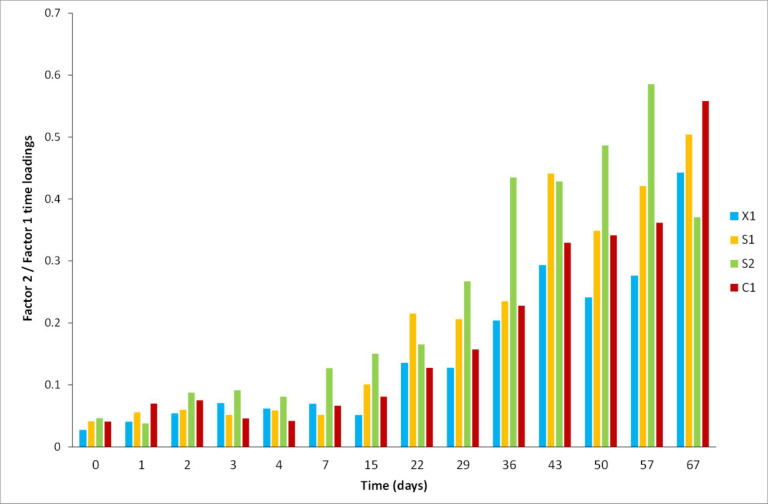
Time-mode PARAFAC loadings of the second to the first-factor ratio for the X1, C1, S1, and S2 cases.

**Figure 10:**
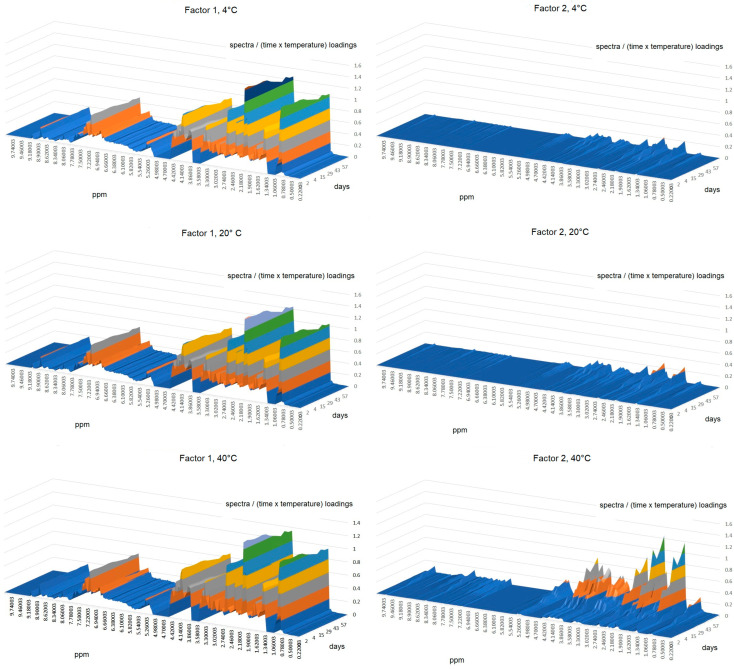
Spectral to time and temperature PARAFAC loadings ratios showing the interaction effect between temperature and time for the S1 case.

Due to the impossibility of disclosing the time effect in a clear way, because of the interactions that this variable has with temperature according to the previous sections and taking advantage of the 3-way data structure, a trilinear modeling strategy was further employed using the PARAFAC algorithm. For the analysis, mode 1 corresponded to time (days), mode 2 to ^1^H-NMR spectra, and mode 3 to temperature. A two-factor model reached 99.66, 99.50, 99.66, and 99.46% of explained variance with residual errors of 4.51, 4.23, 3.32, and 4.26 and core consistencies of 94, 98, 96, and 93% for X1, C1, S1, and S2, respectively. The ratio of the mode 3 (temperature) loading plots (Figure 8) clearly shows similar ratio values at 4 °C and 20 °C for all cases except S2, which significantly differs from those at 40 °C, in accordance with PCA and ASCA modeling. Wines at 40°C varied the most in comparison to the lower ones. The mode 1 (time) loadings ratio (Figure 9) reveals characteristics of temporal variations for each case (C1, X1, S1, and S2), as previously inferred in PCA and ASCA modeling, where it was not possible to identify time variation patterns along the cases. However, it is clear from the plot that, on average, after the seventh day, the sample changes became more abrupt, except for S2, for which the limiting day was the fourth. However, as discussed in the ASCA section, the effect of temperature is not independent, it is case-dependent. As it may be interesting to identify the lapse time in which the integrity of the wine samples is compromised as a function of time and temperature, the mode 2 data were normalized by the others. The results for case S1 are shown in Figure 10, in which each of the normalized factors of the model is plotted at the different studied temperatures. In a sense, this representation is the reconstruction of the spectra in terms of the two factors required for modeling; consequently, it has the advantage that it is easy to track the changes in the spectra due to time and temperature simultaneous variations. In general, the first factor in the PARAFAC model (Factor 1) does not appreciably vary with temperature and can be viewed as a starting point from which further changes can be followed. In contrast, the second factor (Factor 2) has high variation representing the spectra modification along time for each temperature. From Figure 10, it is observed that, in general, at 4 °C, samples remained stable up to for 7 days, at 20 °C for 2 days, and at 40 °C, changes on the first day were already notorious. This indicates that, in general, samples at 20 °C were comparable to those at 4 °C if the lapse time does not exceed 2 days. As expected from previous PCA and ASCA analyses, each case (C1, X1, S1, and S2) presented a characteristic decomposition pattern in this reconstruction. However, the C1, X1, and S2 cases showed similar behavior. These results are in good agreement with those of Jung et al. [21], who demonstrated that a significant temperature influence, calculated as a temperature–time equivalence, needs to be reached to observe the effects.

**Figure 11:**
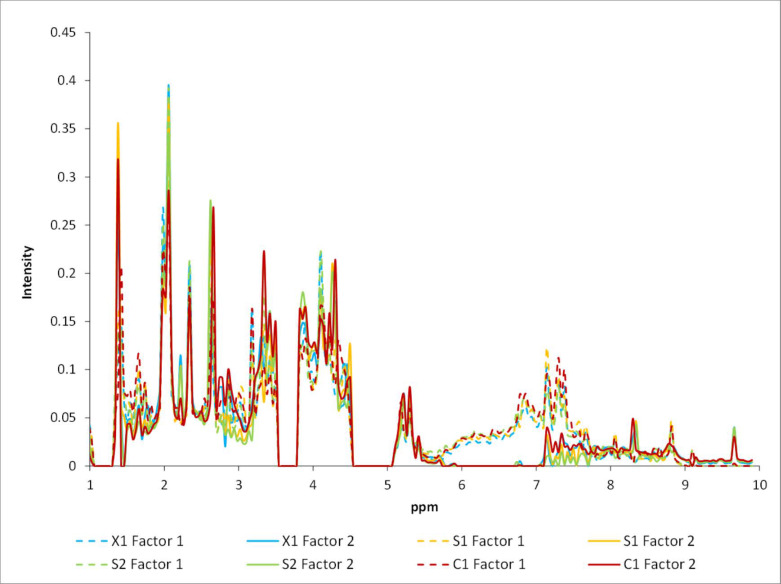
Spectra-mode PARAFAC loadings for the first and second factors for the X1, C1, S1, and S2 cases.

Finally, the mode 2 loadings plot (Figure 11) was used to identify the buckets corresponding to the chemical shifts associated with the observed variations. In general, the buckets listed in Table 2 were identified as those with the major changes. Clearly, each case (C1, X1, S1, and S2) shows a distinctive change pattern in the appearance and disappearance of signals, indicating that the changes in metabolites are case-dependent. This behavior was previously reported by Cassino et al. [8], where wine variations due to aging were observed to be dependent on the type of sample. In addition, it was observed that practically all listed buckets were relevant within each chemometric method, as expected from the fact that the information is the same but processed in a different manner in each chemometric method.

### 2.3. Metabolites Identification

**Figure 12:**
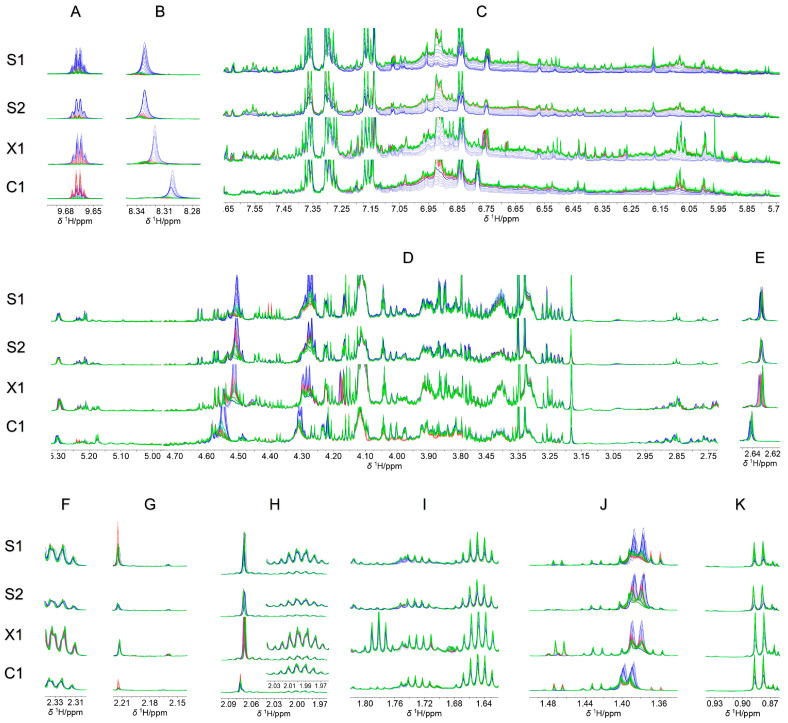
(**A**–**K**) Expansion of regions (bucket list *p* < 0.05) in the overlapped ^1^H-NMR spectra (700 MHz, D_2_O, 300 K). Samples S1, S2, X1, and C1, respectively, stored at 4 °C (green), 20 °C (red), and 40 °C (blue) for 67 days.

The ^1^H-NMR signals contained in the buckets that significantly contribute to describing the changes observed in wine transformation (*p* < 0.05) were assigned to their corresponding metabolites (Table 2). To observe the changes in the signals, ^1^H-NMR spectra expansions of these regions are presented in Figure 12. As shown, acetaldehyde and formic acid raise their concentration with temperature and time increase. This change could be explained by the oxidation of ethanol and methanol. Acetaldehyde is the major oxidation product in wine due to the presence of ethanol. Additionally, this compound could react with anthocyanins and other phenolic compounds [22], which explains the diminishing of the broad signals at *δ* 7.70–5.82 ppm. Acetaldehyde can also be oxidized to acetic acid, increasing its concentration [23]. Furthermore, in the 2.74–5.3 ppm region, some other signals augmented their intensity; however, no metabolite could be assigned to them. Furthermore, the metabolites that diminished their concentration were polyphenols, higher alcohols, a couple of organic acids (lactic and succinic acids), and other non-identified compounds. Higher alcohols can react with organic acids such as lactic and succinic acids, resulting in the formation of new esters [8,24,25]. The reduction in some amino acids (such as alanine, threonine, leucine, and valine) could be due to reactions with the remaining carbohydrates in the wine [26].

## 3. Materials and Methods

### 3.1. Wine Samples

Four red Cabernet Sauvignon commercial wines from different brands obtained from Baja California, Mexico, were analyzed. Their ethanol content varied from 12 to 14% (*v*/*v*). Two Cabernet Sauvignon wines from Valle de Guadalupe, with years of production 2016 (C1) and 2017 (X1), and two from Valle de Santo Tomas, with year of production 2015 (S1 and S2) were analyzed. 

### 3.2. Sample Preparation and Storage Conditions

After the bottles were opened, three aliquots were immediately analyzed to have samples at “zero” time for each wine. At the same time, the rest of the wine samples were transferred to fully-filled conical tubes, covered from any light source, and stored at three different temperatures: 4, 20, and 40 °C for 67 days. The last condition simulated accelerated storage [27]. One fully-filled conical tube was sampled once for each time and temperature condition.

#### Control Samples

Duplicate acquisition of 3 samples of each wine was randomized over a period of 24 min to up to 10 h to simulate the normal time between preparation and acquisition of a set of samples. An average RSD of the sample spectra of 1.4% was determined.

### 3.3. ^1^H-NMR Analysis

For sample preparation, an internal standard solution containing 5.75 mM of 3-(trimethylsilyl) propionic-2,2,3,3-d4 acid sodium salt (TSP, 98% D atom, Sigma-Aldrich, Burlington, MA, USA) in deuterated water (99.9% D_2_O, Cambridge Isotope Laboratories, Andover, MA, USA) was prepared. Then, 900 µL of wine and 100 µL of internal standard solution were transferred into a cryovial and vortex stirred for 30 s; 600 µL of this solution were transferred into a 5 mm NMR tube. No buffer solution was added in order to study wine transformations without the restriction of fixed pH values. The ^1^H-NMR experiments were performed at 300 K on a 700 MHz Avance III HD spectrometer (Bruker, Billerica, MA, USA) equipped with a 5 mm z-axis gradient TCI cryoprobe and a SampleJet autosampler. Data were recorded automatically under ICON-NMR (Bruker, Billerica, MA, USA) control.

^1^H-NMR spectra were acquired with Bruker sequence *noesygppr1d*; water and ethanol signals were suppressed by applying a modulated shaped pulse during a relaxation delay (D1) of 4.0 s and mixing time (D8) of 0.01 s. Each spectrum was acquired with 4 dummy scans (DS), a total acquisition time (AQ) of 3.99 s, 32 scans (NS), and was collected into (TD) 65,536 complex data points and using a spectral width (SW) of 13,992.5 Hz.

### 3.4. NMR Spectra Processing

Free induction decays (FIDs) were Fourier transformed, phased, baseline-corrected, and aligned by shifting the TSP signal to zero, all in automatic mode with 0.3 Hz apodization using TopSpin software v.3.5.6 (Bruker, Billerica, MA, USA).

### 3.5. Multivariate Data Analysis

The ^1^H-NMR data set was reduced by generating homogeneous boxes (binning) of 0.04 ppm in a chemical shift range of 10.00–0.2 ppm, excluding water (5.02–4.70 ppm) and ethanol signals (3.77–3.53, 1.28–1.07 ppm), using alignment and normalization to TSP. Mean center and Pareto scaling were employed for PCA and ASCA analyses. Cross-validation was performed by Venetian blinds with 10 splits and blind thickness of one for both techniques. As for PARAFAC, the spectra were Pareto-scaled before three-way folding, and the spectra mode was non-negativity constrained in the analyses. PLS-Toolbox 9.0 software (Eigenvector Research, Inc., Wenatchee, WA, USA) was employed for all chemometric analyses. 

### 3.6. Metabolite Profiling

^1^H-NMR signal assignment of metabolites was carried out using CHENOMX NMR Suite software v. 8.3 (Chenomx Inc., Edmonton, AB, Canada), consulting the free FooDB database (supported by The Metabolomics Innovation Centre, Edmonton, AB, Canada) [28], and comparing with reported data [2,7,13].

## 4. Conclusions

Chemometric analyses using the PCA, ASCA, and PARAFAC methods were successfully employed in a complementary form to identify relevant characteristics and changes in ^1^H-NMR spectra of opened red wine bottle samples exposed at different temperatures (4, 20, and 40 °C) and time lapses (0 to 67 days) to decide about the sample conditions before further metabolomic studies. Results indicated that changes in temperature and time can satisfactorily be tracked with each chemometric method. PCA analysis revealed variations related to the sample, temperature, and time. The differences in patterns observed comparing each sample indicated that temperature and time effects were case-dependent and that the effect of temperature was not equal at each level of the time variable, i.e., interactions between both variables were relevant. In addition, it showed that the effect of temperature was more discernable than that of time. ASCA analysis was used to take advantage of the full-factorial structure of the experimental data matrix. This chemometric method clearly confirmed: (i) the importance of the interaction between temperature and time in a quantitative way, denoting that the main and interactions effects had comparable magnitudes, (ii) that samples at 4° and 20 °C were more similar between them in comparison to 40 °C, and (iii) that a characteristic profile was present in the samples which define a signature which may be helpful for sample identification purposes, in conditions where the wine has been subjected to temperature and temporal variations. PARAFAC analyses confirmed the results from PCA and ASCA but additionally indicated that, in general, samples at 20 °C were comparable to those at 4 °C if the lapse time does not exceed 2 days. From the buckets associated with their corresponding spectra loadings, identification of the related metabolites was performed. As expected, practically all the listed buckets were relevant to all chemometric methods, as expected from the fact that the information was the same but processed in a different manner in each chemometric algorithm. In general, Acetaldehyde, Formic acid, Acetic acid, Polyphenols, Lactic acid, Methanol, Choline, Succinic acid, Proline, Acetoin, 1,3-propanediol, Isopentanol, Alanine, and other superior alcohols and amino acids were the metabolites responsible for the main changes. It is expected that this information may be useful to evaluate wine quality and integrity when the conditions for the storage and analysis of red wine samples cannot be completely under control due to different field or laboratory situations.

## Figures and Tables

**Table 1:** Metabolites identified in wine samples. Chemical shifts in D_2_O (*δ*), proton multiplicity, coupling constants (*J*), and assignment used for identification are presented.

Peak	Compound	*δ* ^1^H/ppm (Multiplicity, *J* in Hz, Assignation)
1	Acetaldehyde	9.67 (q, 2.98, CH)
2	Trigonelline	9.11 (s, COOH); 8.83 (d, 8.10, CH); 8.80 (d, 6.10, CH); 8.05 (t, CH); 4.42 (s, CH_3_)
3	Formic acid	8.33 (s, CH_3_)
4	Cinnamic acid	7.71 (d, 2 CH_2_)
5	Caffeic acid	7.67 (d, 15.9, CH); 7.2 (d, 2.0, CH); 7.12 (dd, 8.3, 2.0); 6.46 (d, 15.8, CH)
6	Phenethyl alcohol	7.37 (m, CH); 7.30 (m, CH); 3.75 (CH_2_OH); 2.77 (CH_2_)
7	Tyrosine	7.17 (m, 2 CH); 6.84 (m, 2 CH)
8	Gallic acid	7.14 (s, 2 CH)
9	p-Coumaric acid	6.87 (d, 8.4, CH); 6,42 (d, 15.9, CH)
10	Shikimic acid	6.75 (dd, 4.2, 2.2, CH)
11	Fumaric acid	6.64 (s)
12	Epicatechin	6.08 (d, 2.27, CH); 6.06 (d, 2.23, CH)
13	Sucrose	5.43 (d, 3.6, CH); 4.57 (d, 7.7, CH)
14	Arabinose	5.35 (d); 5.32 (d); 5.29 (d, 3.7, CH)
15	Trehalose	5.22 (d, 3.6)
16	Glucose	5.19 (d, 3.6, CH); 4.55 (d, 6.55, CH)
17	Acetoin	4.40 (q, 7.1, CH); 2.2 (s, CH_3_); 1.36 (d, 7.2, CH_3_OH)
18	Ethyl lactate	4.27 (q, 7.0 CH_2_); 4.11 (m, CH_2_); 1.38 (d, 6.9, CH_3_); 1.26 (t, CH_3_)
19	Lactic acid	4.38 (q, 7.0); 1.40 (d, 6.9)
20	Ethyl acetate	4.16 (q, 7.1, CH_2_); 1.24 (t, CH_3_)
21	Myo-inositol	4.04 (t, 3.02, CH); 3.52 (dd, 9.98, 2.85, CH); 3.26 (t, 9.37, CH)
22	Fructose	4.01 (m); 3.98 (m); 3.77 (m)
23	Mannitol	3.86 (dd, 2.9 and 11.8, CH_2_); 3.79 (d, 8.71, CH_2_)
24	Glycerol *	3.77 (m, CH); 3.55 (dd, 11.4 and 6.4, CH_2_)
25	Methanol	3.35(s, CH_3_)
26	Choline	3.18 (s, 3 CH_3_)
27	GABA	2.85 (t, 6.8, CH_2_); 2.42 (t, 7.5, CH_2_); 1.83 (p, 7.7, CH_2_)
28	Succinic acid	2.63(s, 2 CH_2_)
29	Pyuvic acid	2.3(s, CH_3_)
30	Proline	2.3(m, CH_2_); 2.0(m, CH_2_)
31	Acetic acid	2.07(s, CH_3_)
32	1,3-propanediol	1.73 (q, 6.7 Hz, 1H)
33	Isopentanol	1.65 (hept, 6.7, CH); 1.43 (q, 6.94, CH_2_; 0.88 (d, 6.75, 2 CH_3_)
34	Alanine	1.47 (d, 6.5, CH_3_)

* Peaks influenced by ethanol signals suppression.

**Table 2:** Bucket list (*p* < 0.05) with metabolites and not assigned signals related to the major changes in the studied wines.

Figure 12	Bucket (ppm)	Assigned Metabolite
A	9.70–9.62	Acetaldehyde
B	8.34–8.26	Formic acid
C	7.70–5.82	Polyphenols
D	5.30–5.18 4.58–4.46 4.38–4.18 4.14–4.10 4.02–3.78 3.50–2.74	Carbohydrates, Lactic acid, Ethyl lactate, Methanol, Choline, and others
E	2.66–2.62	Succinic acid
F	2.34–2.30	Proline
G H	2.22–2.14 2.10–1.98	Acetoin, Proline, Acetic acid, and others
I	1.82–1.62	1,3-propanediol, Isopentanol, and others
J	1.50–1.34	Alanine, Isopentanol, Lactic acid, and Acetoin
K	0.94–0.86	Higher alcohols and amino acids

## Data Availability

Not applicable.
